# Intermittent Fasting Regulates Metabolic Homeostasis and Improves Cardiovascular Health

**DOI:** 10.1007/s12013-024-01314-9

**Published:** 2024-06-07

**Authors:** Rawan Diab, Lina Dimachkie, Omar Zein, Ali Dakroub, Ali H. Eid

**Affiliations:** 1 Faculty of Medicine, American University of Beirut Medical Center, Beirut, Lebanon; 2 St. Francis Hospital and Heart Center, Roslyn, NY USA; 3 Department of Basic Medical Sciences, College of Medicine, Qatar University, QU Health, Doha, Qatar

**Keywords:** Cardiovascular disease, Metabolic disease, Caloric restriction, Dyslipidemia, Adipose tissue

## Abstract

Obesity is a leading cause of morbidity and mortality globally. While the prevalence of obesity has been increasing, the incidence of its related complications including dyslipidemia and cardiovascular disease (CVD) has also been rising. Recent research has focused on modalities aimed at reducing obesity. Several modalities have been suggested including behavioral and dietary changes, medications, and bariatric surgery. These modalities differ in their effectiveness and invasiveness, with dietary changes gaining more interest due to their minimal risks compared to other modalities. Specifically, intermittent fasting (IF) has been gaining interest in the past decade. IF is characterized by cycles of alternating fasting and eating windows, with several different forms practiced. IF has been shown to reduce weight and alleviate obesity-related complications. Our review of clinical and experimental studies explores the effects of IF on the lipid profile, white adipose tissue (WAT) dynamics, and the gut microbiome. Notably, IF corrects dyslipidemia, reduces WAT accumulation, and decreases inflammation, which reduces CVD and obesity. This comprehensive analysis details the protective metabolic role of IF, advocating for its integration into public health practices.

## Introduction

Obesity is the most prevalent chronic disease worldwide, now estimated to affect over 650 million people globally [1]. The prevalence of obesity and its cardiovascular complications have increased substantially in recent decades to involve younger demographics, with over 340 million children and adolescents aged 5–19 diagnosed as overweight or obese as of 2016 [1]. The World Health Organization (WHO) defines overweight in adults as a body mass index (BMI) of 25 or higher, and obesity as a BMI of 30 or higher. Obesity is highly associated with an array of non-communicable diseases including cardiovascular disease (CVD), type 2 diabetes, dyslipidemia, kidney disease, site-specific cancers, and musculoskeletal disorders [2, 3]. Obesity not only independently increases the risk for CVD but also increases the risk for multiple risk factors associated with CVD such as hypertension, dyslipidemia, and diabetes [4, 5]. Different strategies of obesity prevention have been suggested to reduce its prevalence [1]. Such approaches include dietary changes, medications, and bariatric surgery. These modalities have varied in their effectiveness, with bariatric surgery being the most effective, reducing body weight by 25% to 30%. Glucose-dependent insulinotropic polypeptide (GLP-1) agonists were shown to reduce body weight by 18 to 21%, and behavioral interventions decrease body weight by 5 to 10% [6]. Despite being the most effective, bariatric surgery is the most invasive modality for weight loss and is associated with more complications. In contrast, lifestyle and dietary changes remain the safest and least invasive alternative. Among the different behavioral changes, intermittent fasting (IF) has demonstrated a significant impact on weight loss and could potentially reduce the burden of obesity and dyslipidemia [7].

IF is defined as voluntary cycling between periods of fasting and feeding [8]. Several modalities of IF exist, dependent upon the patterns of eating and fasting windows. The most popular modality is time-restricted eating whereby individuals fast for 16 to 18 h and eat in the remaining 8 or 6 h respectively. In contrast, alternate day fasting (ADF) is a form of IF where individuals alternate between days of restricted and normal eating. On fasting days, caloric intake is limited to 25% of the usual intake, and on the following day, normal eating is resumed [9]. Over the last few decades, IF has emerged as a possible treatment strategy to counter and prevent obesity and cardiovascular disease. IF has been shown to enhance lipid profile, trigger the metabolic switch resulting in weight loss, and modify mitochondrial and oxidative dynamics within cells, ultimately contributing to cardio-protection and longevity. The goal of this review is to highlight the cardioprotective role of IF by providing a comprehensive overview of the effects of IF on cellular dynamics, lipid profile, and adipose tissue concentration. This review strongly advocates for the inclusion of IF in public health recommendations for improved cardiovascular health.

## IF and Lipid Profile

Lipid metabolism is regulated by the interaction of the liver and adipose tissue with different lipoproteins. The liver synthesizes and secretes lipoproteins, namely low-density lipoprotein (LDL), very low-density lipoprotein (VLDL), and high-density lipoprotein (HDL) [10]. HDL acts as a scavenger transporting excess cholesterol from the body to the liver, where it is cleared [11, 12]. VLDL serves to transport triglycerides (TG) to peripheral organs. When VLDL reaches target organs, TG are taken up via lipoprotein lipase (LPL) [13]. This process decreases the proportion of TG in the molecule, transforming it into intermediate-density lipoprotein (IDL), and subsequently into LDL [14]. LDL is mainly composed of cholesterol and will be taken up by cells via specific receptors. The adipose tissue acts as a regulator of fatty acid release and storage and alternates between lipolysis and lipogenesis. This depends on the body’s energy and metabolic demands. At times of high metabolic demand, lipolysis results in the release of free fatty acids into the circulation. These fatty acids then act as a source of energy by transforming into ketone bodies, a process known as beta-oxidation. These ketone bodies then feed into the Krebs cycle resulting in energy in the form of ATP [15]. In times of metabolic sufficiency, fatty acids are used to synthesize TG and enhance adipose tissue’s storage.

The pathogenesis of atherosclerosis, one of the most prevalent cardiovascular diseases, is linked to lipid metabolism. While LDL is mostly taken up by cells in target organs, excess LDL can deposit in the endothelial wall of blood vessels and initiate a cascade that ultimately contributes to the formation of atherosclerotic plaques [16, 17]. These plaques increase in size and may rupture, completely occluding the vessel. This occlusion manifests as cardiovascular events such as myocardial infarction or stroke [18]. The atherosclerotic cardiovascular disease (ASCVD) risk estimator integrates several factors including lipid parameters [19, 20]. By being implicated in the pathophysiology of atherosclerosis and incorporated into ASCVD risk assessment models, lipoproteins are important prognostic indicators of cardiovascular health.

IF has been shown to improve cardiometabolic health by decreasing the levels of total cholesterol, LDL, and TG. ADF decreases TG and LDL levels while increasing LDL particle size [21, 22]. A recent meta-analysis of 35 clinical trials evaluated the effect of IF on lipid profile. Studies with an intervention group of adult patients adhering to an IF diet or energy-restriction diet (ERD) and a control group with at least two weeks of follow-up were included in the analysis. Results of this analysis showed that IF significantly decreases LDL, total cholesterol, and triglycerides [23]. IF decreases TG levels to a greater extent than energy-restriction diets (ERD). However, both ERD and IF have similar effects on LDL levels In contrast, two studies reported no significant change in LDL and TG values with IF [24, 25]. However, the absence of standardization in the timing of lipid value assessments and the selection of metabolically healthy adults with normal baseline LDL values were speculated to explain these findings [24]. The impact of IF on the lipid profile of individuals with normal baseline LDL levels was not pronounced as compared to individuals with elevated LDL at baseline. LDL tends to decrease more significantly in response to IF in individuals with higher baseline levels [26]. In a study evaluating the effects of IF one month after Ramadan, TG levels decreased by 30%, total cholesterol levels increased by 7.9%, and LDL decreased by 11.7% [27]. IF has been shown to decrease LDL levels by 1 to 47 mg/dL, total cholesterol by 5 to 88 mg/dL, and TG by 3–64 mg/dL [28]. Moreover, some trials reported a reduction in LDL levels by 7 to 32% following IF [29]. This is slightly lower and comparable to the decrease in LDL achieved by standard therapy. Low-dose statins were shown to decrease LDL by 20–30% whereas higher doses can decrease LDL levels by 40% [30]. By decreasing LDL, TG, and total cholesterol levels, IF enhances cardiometabolic health and reduces ASCVD risk. Another mechanism by which IF improves the lipid profile is through increasing HDL. IF has been associated with increased HDL levels [25, 31]. This increase is more pronounced when the IF diet is supplemented by an exercise program [25]. In the study evaluating lipid parameters one month after Ramadan, HDL significantly increased by 14.3% [27]. This increase was reproduced in other studies where IF increased HDL by 1 to 14 mg/dl [28]. In contrast, other meta-analyses have found no significant change in HDL levels in response to IF [23, 32]. Despite the controversy, IF may enhance cardiometabolic health by promoting circulating HDL levels.

## IF Mechanism of Action on Lipid Profile

**Fig. 1:**
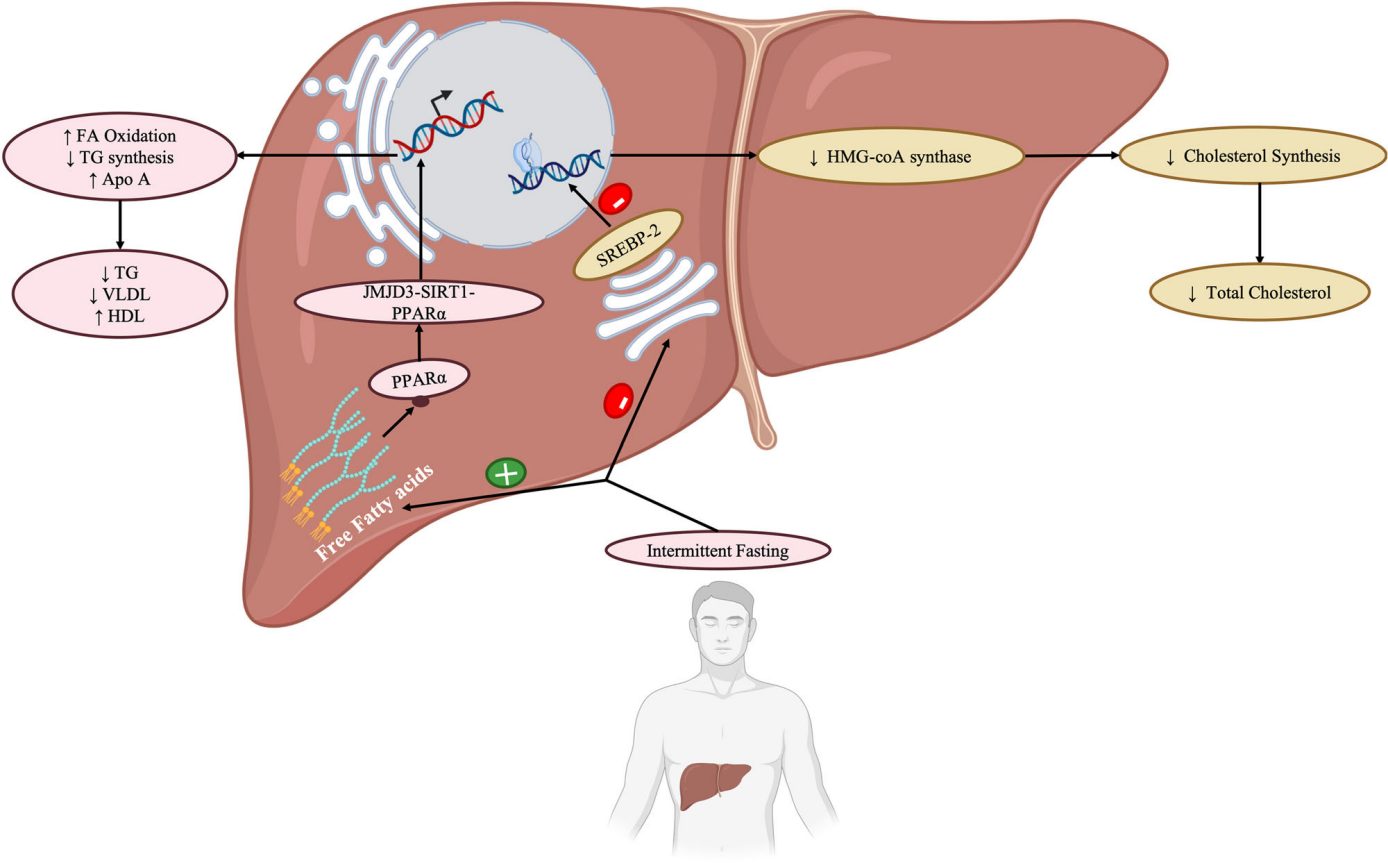
Overview of the metabolic effects induced by intermittent fasting at the cellular level in hepatocytes, highlighting the role of JMJD3-SIRT1-PPAR α complex on lipid and cholesterol metabolism, and the consequential modulation of lipid profile in the fasting state. JMJD3 Jumonji domain-containing protein-3, SIRT1 Sirtuin 1, PPAR α Peroxisome proliferator-activated receptor alpha, FA fatty acid, TG triglyceride, Apo A Apoprotein A, VLDL very low-density lipoprotein, HDL High-density lipoprotein, HMG-coA synthase Hydroxymethylglutaryl-coA synthase, SREBP-2 sterol regulatory element-binding protein 2. This figure was created with BioRender.com

IF confers changes in circulating lipoproteins levels and impacts lipid profile through several mechanisms. IF decreases VLDL, LDL, and TGs by enhancing the expression of proliferator-activated receptor alpha (PPARα) and peroxisome proliferator-activated receptor-gamma coactivator 1-alpha (PGC1α) involved in beta-oxidation [33] (Fig. 1). PPARα and PGC1α are transcription factors involved in lipid metabolism and implicated in the pathogenesis of dyslipidemia [34]. PPAR α and PGC1a play key roles as regulators in the cardioprotective effect of IF. IF increases the expression of PPAR α and PGC1a in hepatocytes [35]. The upregulation of PPAR α and PGC1 α results in the activation of fatty acid oxidation by promoting fatty acid uptake and transport as well as the transcription of genes involved in peroxisomal oxidation [36, 37]. This increase in fatty acid oxidation decreases TG storage in adipose tissues and subsequently leads to lower production of VLDLs by the liver [38]. The decrease in VLDLs is accompanied by a decrease in LDL and small and dense LDL (sdLDL), which are the main culprits in the pathophysiology of atherosclerosis [35]. In addition, fasting in PPAR α - null mice resulted in fatty liver disease which was attributed to a decrease in fatty acid oxidation evidenced by low levels of plasma hydroxybutyrate [33, 39, 40]. This further strengthens that IF exerts its effects on TG, VLDL, sdLDL, LDL, and fatty acid oxidation through upregulation of PPAR α in hepatocytes.

IF increases HDL by enhancing the synthesis of apoprotein A (ApoA) and downregulating cholesteryl ester transfer protein (CETP). IF has been shown to increase the production of apoprotein A by the liver [41–44] (Fig. 1). The increase in ApoA promotes an increase in HDL assembly and production. In addition, IF reduces the expression of the CETP [45, 46]. CETP regulates the translocation of cholesterol esters from HDL to VLDL [47]. By reducing CETP, IF inhibits HDL conversion and subsequently enhances HDL levels while decreasing VLDL.

IF reduces total cholesterol synthesis by decreasing the expression of sterol regulatory element binding protein-2 (SREBP-2) [48]. SREBP-2 is a transcription factor that binds to sterol regulatory element (SRE) in the enhancer region that promotes several enzymes’ transcriptions [49, 50] (Fig. 1). These enzymes include HMG-coenzyme A synthase, HMG-coenzyme A reductase, and squalene synthase [51]. By decreasing SREBP-2, IF downregulates the mRNA level of these enzymes, which results in decreased cholesterol synthesis by the liver [48].

IF has demonstrated great potential in correcting dyslipidemia by increasing HDL and decreasing LDL, VLDL, TG, and total cholesterol through various mechanisms. The proposed mechanisms explain the observed alterations in lipid parameters induced by IF.

## IF and Lipid Metabolism

IF induces beta-oxidation and leads to increased ketone production which serves as a crucial fuel source for the body. The substantial role of ketones is even more prominent during periods of fasting, as they provide energy for vital organs particularly the brain [52]. Around 12 to 36 h after the last meal, the body enters the third phase of fasting and starts to utilize lipids for energy production instead of glycogen, a phenomenon known as the “metabolic switch”. IF appears to promote the metabolic switch through the breakdown of TGs in adipose tissue into fatty acids [53]. These free fatty acids are then transported to the liver where they undergo beta-oxidation to produce ketones. Ketones are metabolized inside cells into acetyl-CoA, which enters the Krebs cycle and generates large amounts of ATP [54].

**Fig. 2:**
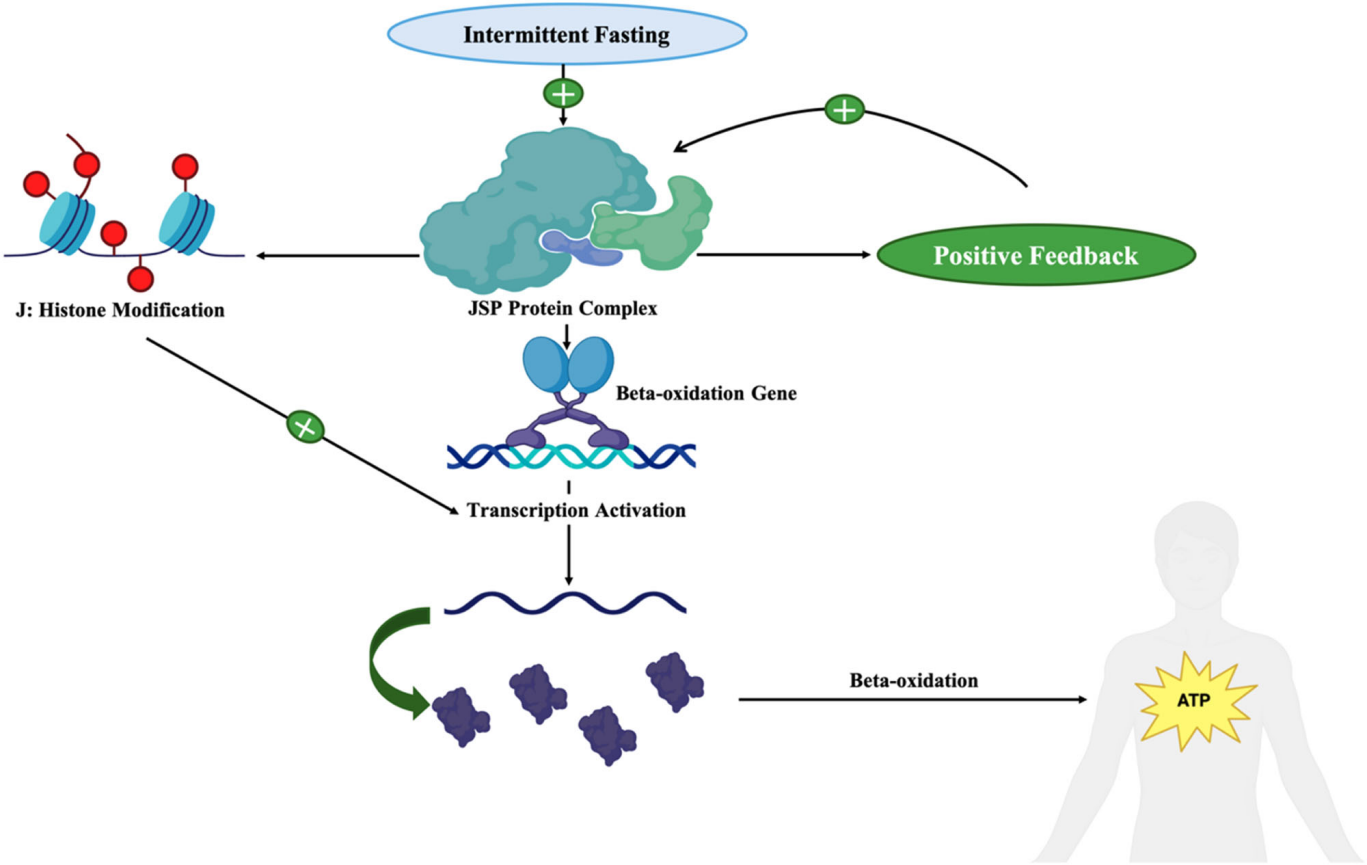
A flowchart depicting the activation of the JMJD3-SIRT1-PPAR α complex, which enhances beta-oxidation and provides energy to the individual during intermittent fasting. Once activated, the JSP complex activates itself via a positive feedback mechanism and acts via histone modification to promote the transcription of genes involved in beta-oxidation, resulting in ATP formation. JSP complex: JMJD3-SIRT1-PPAR α complex; JMJD3 Jumonji domain-containing protein-3, SIRT1 Sirtuin 1, PPAR α Peroxisome proliferator-activated receptor alpha, ATP adenosine triphosphate. This figure was created with BioRender.com

IF promotes beta-oxidation through the upregulation of the expression of the protein complex JMJD3-SIRT1-PPARα [55] (Fig. 2). This protein complex stimulates the expression of beta-oxidation genes [56]. Moreover, this complex leads to a positive feedback loop that promotes its expression, further stimulating beta-oxidation [57]. By enhancing the expression of JMJD3-SIRT1-PPARα, IF significantly increases beta-oxidation and the generation of ketones.

JMJD3, the first mediator in the complex, promotes epigenetic modifications that facilitate the increase in beta-oxidation during IF. JMJD3 acts as a histone demethylase that specifically targets histone H3 on lysine 27 (H3K27) [56]. Many genes involved in beta-oxidation bear both methylated H3K4 and H3K27 which results in a bivalent marking that regulates overall gene expression [58]. The methylation of H3K27 represses gene expression whereas the methylation of H3K4 activates gene expression [58]. By demethylating H3K27, JMJD3 reverses the gene silencing effect, allowing methylated H3K4 to facilitate gene transcription [58, 59]. IF increases beta-oxidation during fasting through the upregulation of JMJD3 expression which contributes to the bivalent epigenetic modification of histones.

On the other hand, SIRT1 and PPARα act synergistically to upregulate beta-oxidation during IF. SIRT1 modulates lipid metabolism in the liver through the upregulation of PPARα [60]. As discussed in the previous section, PPARα is a transcription factor that promotes the expression of several genes implicated in beta-oxidation [61–63] (Fig. 2). These genes include those involved in fatty acid transport into the mitochondria (Cpt1, Cpt2, Slc25a20, Slc22a5), acyl-CoA dehydrogenases, and other genes involved in beta-oxidation of unsaturated fatty acids [55]. Moreover, PPARα activates HMG-CoA synthase and HMG-CoA Lyase which regulate ketogenesis [55]. PPARα also governs the expression of genes that encode flavoproteins and dehydrogenases. This group of proteins is responsible for the transport of electrons from acyl-CoA dehydrogenases into the oxidative phosphorylation pathway, which ultimately generates ATP from the acyl-coA [55]. By enhancing the expression of the SIRT1-PPARα axis, IF increases fatty acid oxidation and allows the production of energy during fasting.

## Effects of IF on Oxidative Stress and Longevity

**Fig. 3:**
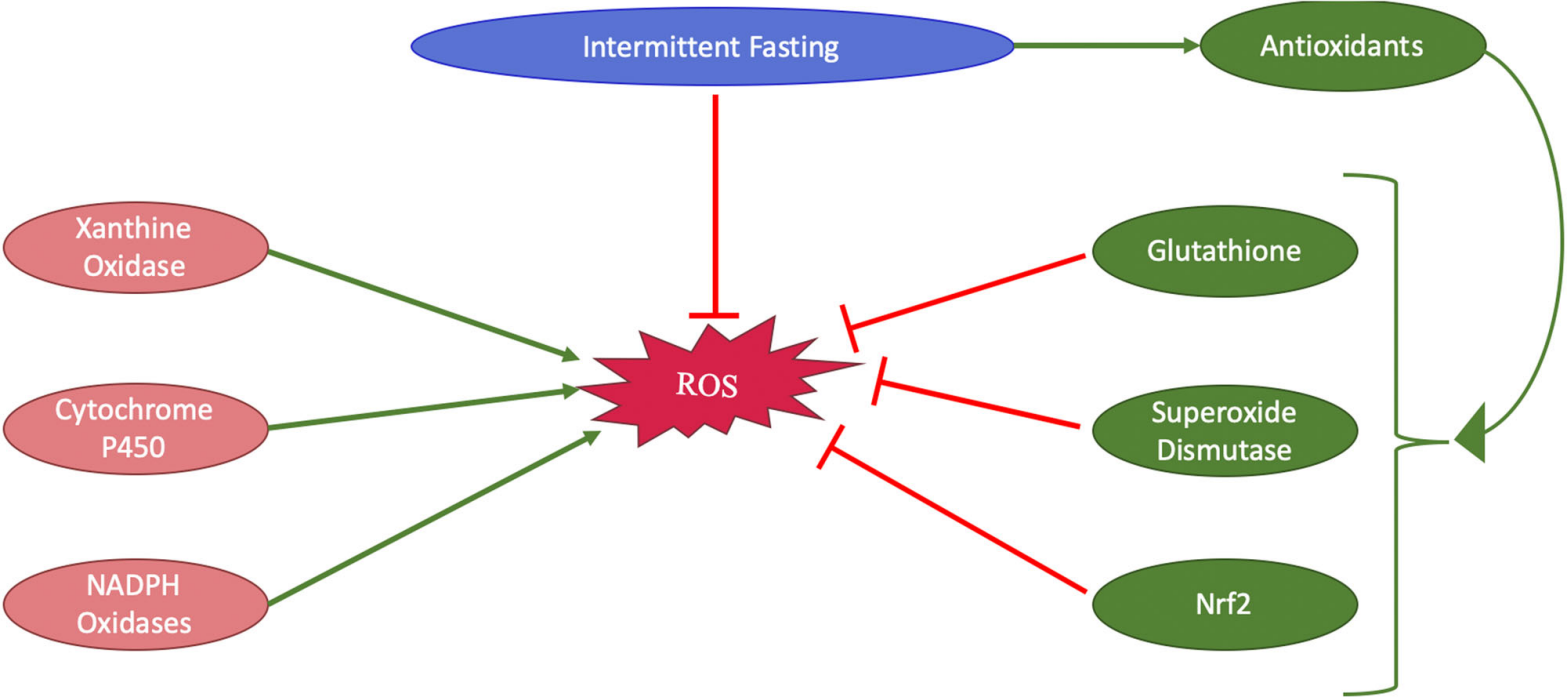
A flowchart depicting the protective effect of intermittent fasting on oxidative stress by promoting antioxidants including glutathione, superoxide dismutase, and Nrf2 that decrease the generation of ROS. On the other hand, enzymes such as Xanthine oxidase, cytochrome p450, and NADPH oxidases contribute to the generation of ROS. Nrf2 nuclear factor erythroid 2-related factor 2, ROS reactive oxygen species, NADPH Nicotinamide adenine dinucleotide phosphate hydrogen

Oxidative stress results from an imbalance between reactive oxygen species (ROS) and antioxidants in cells. ROS, including free radicals like superoxide and hydrogen peroxide, are primarily produced in the mitochondria during aerobic metabolism [64]. Elevated ROS levels or reduced antioxidant defenses lead to oxidative stress which results in damage to biomolecules such as proteins, lipids, carbohydrates, and nucleic acids [65, 66]. For instance, lipids form compounds like malondialdehyde (MDA) as a result of peroxidation. Protein oxidation disrupts normal redox signaling, which is crucial for cellular communication and function. This damage results in the onset of diseases, including cancer which is precipitated by mutations from oxidative damage [67]. Antioxidants help reduce oxidative stress by preventing ROS formation, blocking ROS from causing cellular damage, and repairing any damage that occurs. These antioxidants include enzymes like superoxide dismutase (SOD), which neutralize ROS into attenuated forms. Glutathione (GSH), another antioxidant, acts as a potent defender by directly neutralizing ROS and supporting other antioxidant enzymes. Moreover, another component of the antioxidant cascade, known as nuclear factor erythroid 2-related factor 2 (Nrf2), plays a crucial role in controlling antioxidant production (Fig. 3). When the cell senses oxidative stress, Nrf2 responds by translocating from the cytoplasm to the nucleus, where it binds to the antioxidant-response elements located in the promoter regions of genes responsible for antioxidant production [68, 69]. This balance between antioxidants and ROS is essential for cellular health and overall well-being.

**Fig. 4:**
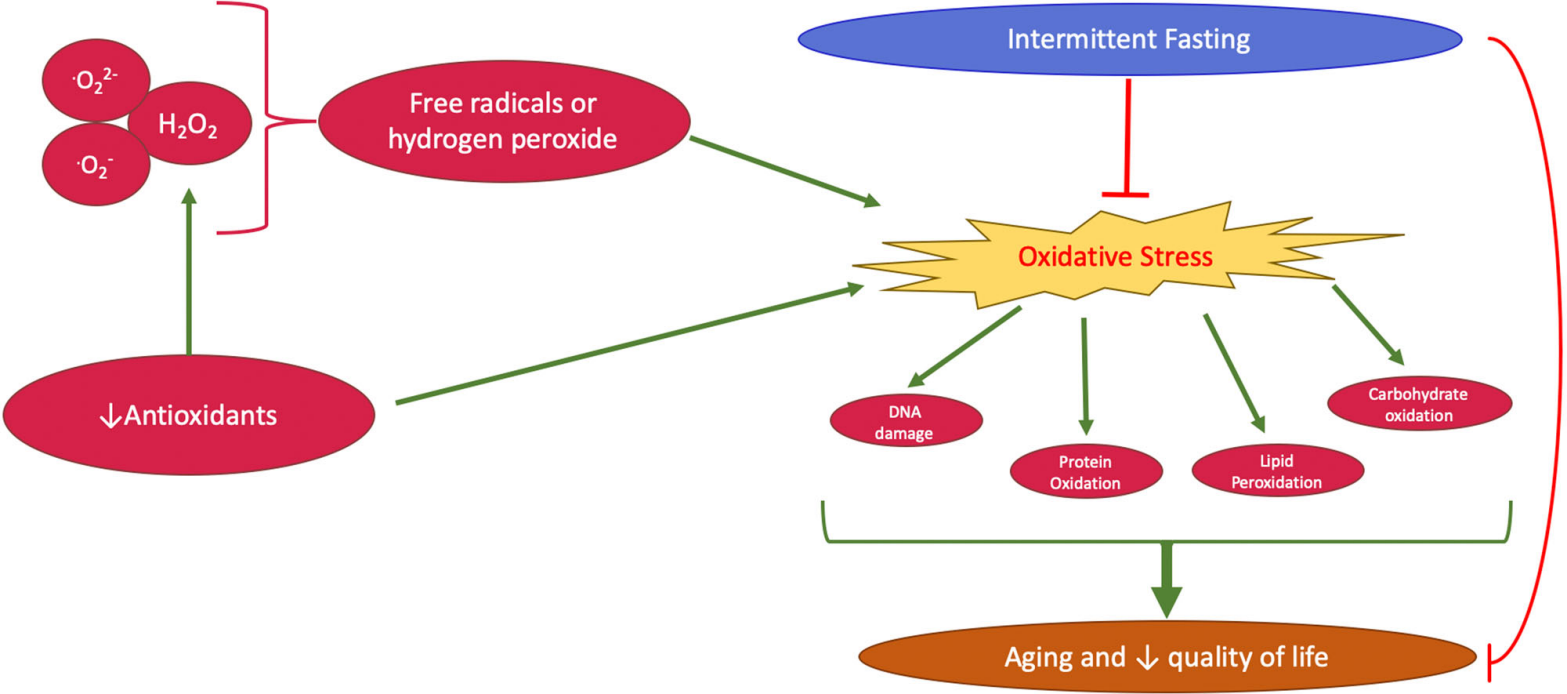
A flow chart illustrating the protective role of intermittent fasting on oxidative stress and longevity. IF reduces oxidative stress which leads to a decrease in oxidative stress-mediated DNA damage, protein oxidation, lipid peroxidation, and carbohydrate oxidation, which contributes to longevity and better quality of life

IF shows significant promise in reducing oxidative stress and promoting longevity (Fig. 4). IF enhances the body’s antioxidant defenses and has been associated with reductions in markers of oxidative stress such as serum MDA and an increase in GSH levels during fasting periods [70] (Fig. 3). IF regimes, including time-restricted feeding (TRF) and alternate-day fasting (ADF), have been associated with the upregulation of protective enzymes like Nrf2 and SOD2. A study including overweight males engaging in IF for two days per week without any caloric restriction for a total of 12 weeks showed significant health benefits. In this study, participants consumed a light meal before sunrise and a full meal after sunset during the two fasting days per week with an intake reduction of 300–500 calories daily. This dietary lifestyle was associated with a notable decrease in plasma MDA and DNA damage and an average weight loss of approximately 2.5 kg compared to the control group [71]. Similarly, a study on obese prediabetic men demonstrated that early time-restricted feeding (eTRF) significantly decreased plasma 8-isoprostane levels, a marker of oxidative stress, by roughly 14%. This change was attributed to an increase in the control group’s levels, while the eTRF group’s levels stayed consistent. This study differs from other studies since the investigators provided strict diet control by providing all meals for the participants and monitoring meal consumption [71]. Moreover, prolonged nighttime fasting was found to enhance total antioxidant capacity without altering daily caloric intake or macronutrient composition [72]. Combining moderate physical activity with IF significantly reduced markers of inflammation while supporting antioxidant function [73]. IF was also shown to reduce oxidative stress in organs like the heart and brain [74]. Some studies link the reduction in oxidative stress during IF to weight loss and emphasize that reductions in oxidative stress markers may not occur unless accompanied by a decrease in body weight [75]. Taken together, these reports argue that IF plays a crucial role in enhancing health and longevity by attenuating oxidative stress and improving brain function (Fig. 4).

## IF and Cardiac Physiology

Cardiovascular diseases (CVD) are a leading cause of global mortality, accounting for approximately one-third of all deaths worldwide [76]. Risk factors for CVD can be categorized as unmodifiable: age, gender, genetics, and modifiable: lifestyle factors like smoking, obesity, sedentary behavior, dyslipidemia, hypertension, diabetes, and poor diet. Prevention and treatment strategies focus on lifestyle modifications, alongside pharmacotherapy and invasive interventions when necessary [9]. Emphasizing preventive measures and personalized treatment plans underscores the importance of lifestyle changes in reducing the burden of CVD globally.

IF has emerged as a potential strategy for reducing CVD risk factors [9, 46, 77]. IF promotes metabolic shifts from glucose to fatty acids and ketones, resulting in improved lipid profiles with reductions in total cholesterol, triglycerides, and LDL cholesterol levels [9, 77, 78]. IF inhibits atherosclerotic plaque development by decreasing inflammatory markers and prevents monocyte adhesion to vascular endothelial cells, limiting atherosclerosis progression [9]. Additionally, IF increases brain-derived neurotrophic factor (BDNF) and leads to a reduction in blood pressure [9, 77, 78]. It aids in obesity and diabetes management by promoting weight loss, enhancing glucose metabolism, and improving insulin sensitivity [9, 46]. IF also mitigates cardiac hypertrophy and failure and improves right ventricle function in patients with pulmonary arterial hypertension [9]. Moreover, IF may offer cardiac protection post-cardiovascular events, as evidenced by lower rates of acute decompensated heart failure during Ramadan among individuals with pre-existing ischemic heart disease. Overall, these findings highlight the diverse benefits of IF in cardiovascular health, advocating for its inclusion in comprehensive strategies against CVD.

IF may improve cardiovascular outcomes through various mechanisms. One mechanism is through the “oxidative stress hypothesis”, which suggests that IF reduces oxidative stress by limiting free radical production and decreasing mitochondrial energy output [77]. Another mechanism involves the circadian rhythm theory, which emphasizes synchronizing eating patterns with circadian clocks to optimize glucose and fat metabolism [77]. In addition, IF also promotes a ketogenic state, leading to lowered blood pressure and reduced fat accumulation [77, 78]. These pathways suggest that IF could be a valuable tool in managing CVD risk factors and informing novel dietary recommendations for cardiovascular health maintenance.

## IF and Mitochondrial Dynamics

IF positively modulates mitochondrial dynamics resulting in preserved mitochondrial health and cellular energetics. Mitochondrial dynamics play a crucial role in cellular homeostasis and include various aspects of mitochondrial function, including morphology, distribution, turnover, and quality control mechanisms such as mitophagy. Mitochondrial health declines with age, and this decline is more pronounced in highly oxidative tissues such as skeletal muscles. Studies have shown that intermittent fasting selectively modulates mitochondrial dynamics in ways that promote mitochondrial health and functionality [79, 80].

Several studies have proposed mechanisms underlying the effect of IF on mitochondrial health. In a study conducted on mice, time-controlled fasting was found to prevent aging-like mitochondrial changes induced by persistent dietary fat overload in skeletal muscle. This preservation of mitochondrial integrity may be attributed to the suppression of excessive mitochondrial fission by the upregulation of adipose triglyceride lipase Atgl, the rate-limiting enzyme of triglyceride hydrolysis. The liberation of fatty acids by Atgl during fasting contributes to enhanced mitochondrial oxidative capacity [81]. In addition, IF promotes the fusion of mitochondria through the upregulation of Sirtuin 3 expression [82]. This in turn leads to the deacetylation of Malate dehydrogenase 2 (MDH2) promoting mitochondrial fusion. Another mechanism by which IF regulates mitochondrial dynamics is through the induction of mitophagy [83]. Mitophagy is the process by which damaged or dysfunctional mitochondria are selectively removed via autophagy. Caloric restriction, a key component of intermittent fasting, has been shown to stimulate mitophagy pathways, leading to the clearance of dysfunctional mitochondria and the promotion of mitochondrial turnover. This process is crucial for preventing the accumulation of damaged mitochondria, which can compromise cellular energetics and contribute to age-related decline. Moreover, IF mediates epigenetic changes by promoting histone acetylation in the promoter regions of mitochondrial activating genes in muscle tissues. This enhances the expression of genes involved in mitochondrial function and energy metabolism [84]. The effect of IF on mitochondrial dynamics was potentiated by exercise. In a study comparing two groups of mice subject to every-other-day intermittent fasting with or without high-intensity exercise, exercise was shown to increase mitochondrial mass compared to the control group. The exercise and IF group also had a lower level of oxidative stress and greater oxidative capacity compared to the IF group alone [85]. By promoting a balanced fusion-fission equilibrium and inducing mitophagy, IF contributes to preserved mitochondrial health and cellular function.

Prolonged intermittent fasting modulates mitochondrial functions in neurons, leading to neuroprotective effects against aging and neurodegenerative diseases [86]. The decline in mitochondrial function with age is a proposed mechanism for many neurodegenerative diseases, owing to the role of mitochondria in preventing oxidative damage. Intermittent fasting was shown to promote neuroprotection by its effects on mitochondrial function. IF results in the generation of ketone bodies including beta-hydroxybutyrate, which act as enhancers of mitochondrial function. In a recent study conducted on neurons in vitro, the addition of beta-hydroxybutyrate to the medium was shown to enhance mitochondrial function and this enhancement was more pronounced in conditions of prolonged fasting for more than 12 h [87]. In this experiment, the activity of citrate synthase, the first enzyme in the tricyclic acid cycle, was measured as an indicator of mitochondrial function. Mitochondrial function was also assessed by flow cytometry in the same study. Beta-hydroxybutyrate was shown to bind receptors in the brain, heart, and liver where it enhances mitochondrial function [87]. It activates monocarboxylic acid transporter 1 (MCT-1) and succinyl-coA transferase in the heart, enzymes involved in ketolysis [87]. IF enhances mitochondrial respiratory control rate, resulting in energy production that supports neurons during fasting. By doing so, IF modulates mitochondrial bioenergetics and oxidative stress response pathways in the brain, promoting neuronal survival and cognitive function during aging and neurodegeneration.

## IF and Epigenetics

IF has emerged as a potent modulator of DNA methylation patterns, a key epigenetic mechanism. Rodents subjected to caloric restriction (CR), a form of IF, underwent significant alterations in DNA methylation, particularly in genes associated with aging-related pathways [88, 89]. CR-induced changes in DNA methylation involve both hypomethylated and hypermethylated regions. Furthermore, even short-term CR interventions have been shown to reverse age-related alterations in DNA methylation. Interestingly, maternal IF has been shown to significantly decrease levels of DNA methyltransferase in offspring, suggesting a potential intergenerational impact on DNA methylation dynamics [89]. By modulating DNA methylation, IF may contribute to the beneficial effects on metabolic health and longevity.

IF modulates gene expression through histone modifications and the regulation of small noncoding RNAs, particularly microRNAs (miRNAs). Histone methylation is significantly influenced by IF, particularly in metabolic switching processes. IF was found to induce differential modulation of the H3K9 trimethylation (H3K9me3) mark [90]. These epigenetic modifications induced by IF persist even after the resumption of normal feeding patterns, indicating a sustained impact on the chromatin landscape. Moreover, genes affected by H3K9me3 modifications in response to IF are implicated in metabolic pathways, suggesting a direct link between histone methylation and metabolic regulation [90]. Additionally, caloric restriction can counteract age-dependent increases in specific miRNAs associated with apoptosis, including miR-181a-1*, miR-30e, and miR-34a, to promote cellular survival [88]. By downregulating these miRNAs, IF enhances the expression of their target genes, including those involved in apoptosis regulation and neuronal survival, contributing to its neuroprotective effects [88, 91]. Thus, IF, through its influence on histone modifications and miRNA regulation, plays a role in promoting cellular survival and neuroprotection.

In addition, IF influences the epigenome through its interaction with autophagy-induced chromatin remodeling. Autophagy is a cellular process crucial for maintaining cellular homeostasis and is triggered by nutrient deprivation, such as fasting. Autophagy has been linked to alterations in DNA methylation and post-translational histone modifications including histone H4 lysine 16 acetylation (H4K16ac) which regulate cellular lifespan and survival responses [91]. The modulation of these histone marks by IF-induced autophagy represents a key mechanism by which fasting influences the epigenome, potentially contributing to its beneficial effects on health span and longevity across generations [89, 91].

IF exerts various effects on epigenetic regulation by influencing gene expression patterns and chromatin modifications. Through its modulation of DNA methylation, histone modifications, and non-coding RNAs, IF impacts individual health and extends its effects across generations to impact aging, metabolism, and longevity.

## IF and White Adipose Tissue

By its role in white adipose tissue (WAT) browning, IF reduces the complications of WAT accumulation in the body and decreases body weight. WAT is distinct from brown adipose tissue (BAT) and serves primarily as a lipid storage depot [92]. However, WAT can acquire similar features to BAT through a process known as “browning” [92]. The inhibition of WAT browning has been well correlated with an increase in obesity rates [93]. Consequently, the promotion of browning is emerging as a novel strategy to combat obesity. Browning of WAT can be induced by various exogenous stimuli [94]. IF has been demonstrated to enhance WAT browning and improve metabolic profiles [95–98]. IF-induced WAT browning is a promising strategy to alleviate complications associated with WAT accumulation, including inflammation and hypoxia.

IF has been shown to promote beneficial effects by suppressing the inflammatory state induced by WAT accumulation. Inflammation arises when there is a profuse activation of immune cells like macrophages [92]. The accumulation of WAT promotes the switching of anti-inflammatory M2 macrophages to pro-inflammatory M1 macrophages, exacerbating the inflammatory response [99]. Adiponectin, an adipocytokine with key metabolic effects, counteracts this switch [100]. However, adiponectin levels are reduced in situations like obesity, contributing to an increased M1 to M2 ratio [100]. This imbalance leads to the infiltration of M1 macrophages, forming CD11c+ crown-like structures (CLS) around dead cells in WAT, resulting in the accumulation of high amounts of lipids within macrophages and promoting inflammation [99, 101]. Moreover, these CD11c+ macrophages release pro-inflammatory markers such as TNF-a, IL-6, C-reactive protein (CRP), and monocyte chemotactic protein-1 (MCP-1), all of which exacerbate inflammation (55; 59). Additionally, macrophages enhance ECM deposition which increases the release of proinflammatory cytokines and results in a vicious cycle that perpetuates the inflammatory state [97]. IF has been shown to enhance the gene expression of certain M2 macrophage markers, partly through increasing adiponectin levels [95, 99]. Moreover, IF decreases the number of several markers of inflammation in adipose tissue such as Lgals3, Itgax, and Ccl2, and stimulates the degradation of ECM [97, 102]. Therefore, IF reduces the inflammation induced by WAT accumulation by promoting the switch to M2 macrophages, preventing excess deposition of ECM, and inhibiting the massive release of proinflammatory cytokines.

In addition, IF has been shown to decrease hypoxia-induced damage caused by WAT. WAT accumulation results in an increased demand for nutrients and oxygen to sustain proliferating adipocytes. Although the body attempts to increase the supply of nutrients by stimulating the production of new blood vessels through angiogenesis, these efforts often fall short. This results in a supply-demand mismatch leading to hypoxic adipocytes [103, 104]. Interestingly, IF has been shown to alleviate hypoxia by stimulating angiogenesis through increasing the expression of VEGF-A. [92]. VEGF-A promotes angiogenesis, exhibits anti-inflammatory properties, and induces WAT browning through the activation of the sympathetic nervous system [105]. IF reduces hypoxia and inflammation caused by WAT accumulation by upregulating VEGF-A expression.

## Intermittent Fasting and Gut Microbiome

IF can contribute to several cardioprotective effects by altering the composition of the gut microbiome. The gut microbiome has been extensively studied for its role in disease pathogenesis and protective immunity. The composition of the gut microbiome is linked to the host’s overall health. For instance, changes in microbiome composition can trigger molecular cascades that contribute to atherosclerosis, dyslipidemia, and obesity [106]. IF plays a multifaceted role in shaping the gut microbiome and favorably modulating metabolic health. IF decreases the ratio of firmicutes to Bacteroidetes and increases the abundance of *Allobaculum* and *Bifidobacterium* [107]. Through its effects on the microbiome composition, IF enhances epithelial barrier function, prevents inflammation, and promotes WAT browning [96, 107, 108].

IF lowers the risk of systemic inflammation and atherosclerosis by preventing the leakage of lipopolysaccharide (LPS) endotoxin into the bloodstream. LPS endotoxin is a virulence factor secreted by many bacterial species, and its presence systemically is referred to as endotoxemia. LPS activates receptors on macrophages and other cells of the innate immune system, resulting in the release of cytokines and further leukocyte recruitment. This cytokine storm is at the origin of systemic and chronic inflammatory processes, which predispose to many diseases, especially cardiovascular diseases and dyslipidemias [106]. Systemic inflammation may contribute to the progression of atherosclerosis in a previously susceptible individual. By decreasing intestinal permeability, IF decreases the leakage of LPS as well as other antigens that might trigger the immune system. This protects against atherosclerosis, insulin resistance, obesity, and type 2 diabetes [109]. IF protects against low-grade chronic inflammation and its induced conditions by enhancing intestinal barrier function and preventing LPS leakage.

The protective role of IF during inflammation in enhancing intestinal barrier function is accomplished through two main mechanisms. IF first increases the abundance of *Allobaculum*, a genus known to synthesize short-chain fatty acids (SCFAs) such as butyrate, propionate, and acetate [107]. SCFAs are known for their role in maintaining the intestinal barrier. They induce a “physiologic hypoxia” state by promoting epithelial cell metabolism that consumes oxygen [110]. This hypoxic state stabilizes hypoxia-inducible factor (HIF-1), a crucial transcription factor mediating the protective effect of SCFAs. Other mechanisms that act synergistically include histone deacetylase inhibition and stimulation of GRP43 and GRP109a [108]. However, the stabilization of HIF-1 remains the major mechanism by which SCFAs act [111]. HIF-1 activates human intestinal trefoil factor (ITF) [112]. ITF is a small peptide found abundantly throughout the gut and known to concentrate at sites of injury, such as in proximity to peptic ulcers [113]. The HIF-1-ITF axis maintains the intestinal epithelial barrier and decreases inflammation. Another mechanism by which IF reduces intestinal permeability is by increasing the abundance of *Bifidobacterium* in the gut. *Bifidobacterium* is a probiotic, living, non-pathogenic bacterium involved in intestinal protection [114]. *Bifidobacterium* decreases Zonulin, a protein that increases intestinal permeability by disrupting tight junctions through a protein kinase C-dependent mechanism [115]. *Bifidobacterium* also decreases the release of inflammatory cytokines [115]. Hence, *Bifidobacterium* improves intestinal barrier function through the downregulation of both Zonulin and inflammatory cytokines. IF contributes to the maintenance of the intestinal barrier during inflammation by increasing *Allobaculum and Bifidobacterium*.

IF promotes WAT browning and BAT differentiation by increasing the ratio of Firmicutes to Bacteroidetes. IF increases the abundance of firmicutes, the main contributors to acetate in the gut. Acetate is one of the SCFAs produced during fasting [116]. Acetate promotes browning by increasing the expression of PRDM16, UCP1, DIO2, and PGC-1, which are genes implicated in adipose browning [117]. These genes contribute to WAT browning by increasing energy expenditure in adipose tissue and by increasing mitochondrial synthesis. PGC-1 enhances the expression of nuclear respiratory factor NRF-1, which binds the promoter region of mitochondrial transcription factor A (mtTFA). mtTFA in turn triggers mitochondrial replication and transcription-promoting energy expenditure [118].

By its role in altering the composition of the gut microbiome, IF decreases intestinal permeability and initiates WAT browning. The decrease in permeability improves barrier function, thereby preventing the leakage of LPS into the bloodstream. This reduces the risk of chronic inflammation and associated cardiometabolic diseases.

## IF and Potential Drawbacks

Despite the numerous benefits of IF on cardiovascular health and weight loss, it is important to recognize the potential drawbacks of this dietary regimen. First, research has shown that IF can lead to nutritional deficiencies due to reduced intake during fasting periods [53, 119]. Human studies have reported associations between intermittent fasting and decreased intake of key nutrients, such as calcium, vitamin D, and essential fatty acids [120, 121]. Second, individuals with certain medical conditions, such as diabetes, may experience disruptions in blood sugar levels, posing risks to metabolic health [122]. Alternatively, some studies have proposed that IF can help with diabetes since it improves insulin sensitivity [123]. Additionally, IF may trigger or exacerbate binge eating episodes and restrictive behaviors in susceptible individuals, including those with a history of eating disorders such as anorexia nervosa, bulimia nervosa, or binge eating disorder [123–125]. The rigid meal timing and prolonged fasting periods characteristic of some IF protocols can reinforce unhealthy eating patterns and exacerbate feelings of guilt, shame, or loss of control surrounding food. Moreover, severe caloric restriction or prolonged fasting, both of which are components of the IF regimen, can affect the hypothalamic-pituitary-gonadal (HPG) axis leading to dysregulation in the release of reproductive hormones, namely estrogen and progesterone. This leads to irregular menstrual cycles or the absence of menstruation in women [123, 126]. This was demonstrated in experiments on female rats where IF affected the estrous and led to delayed ovulation timing and irregular levels of reproductive hormones. This was also reproduced in human studies, where IF prolonged the menstrual cycle. These irregularities are hypothesized to arise from irregular levels of reproductive hormones and changes in insulin sensitivity and leptin levels triggered by fasting regimens [123]. Psychological effects of IF include increased feelings of hunger, irritability, and difficulty concentrating during fasting periods, potentially impacting daily functioning [127, 128]. These findings highlight the importance of considering individual circumstances and consulting with healthcare professionals before adopting IF as a dietary approach.

## Conclusion

Obesity remains a major cause of cardiovascular disease worldwide. Lifestyle modifications are a cornerstone in the management of obesity. Even though caloric restriction is the most recommended regimen for weight loss, it often fails due to a lack of compliance. IF serves as a promising alternative to the harsh lifestyle of caloric restriction. IF has been shown to modulate lipid profile, enhance lipid metabolism, reduce the risk of cardiovascular diseases, and prevent inflammation. IF is emerging as a potential treatment strategy to counter obesity and enhance cardiometabolic health.

Further investigations and randomized controlled trials are needed to elucidate the effects of IF on metabolic health in specific target populations such as people with diabetes. Moreover, randomized controlled trials with longer follow-up durations are required to enhance our understanding of the long-term effects of IF, the sustainability of this dietary regimen, and the potential side effects of IF. Lastly, there are a minority of studies focusing on the effects of IF on metabolic health irrespective of weight loss, and more studies are needed to further elucidate whether IF could be beneficial in the absence of weight loss.
